# Significant incidental cardiac disease on thoracic CT: what the general radiologist needs to know

**DOI:** 10.1186/s13244-019-0693-y

**Published:** 2019-02-06

**Authors:** Maren Krueger, Paul Cronin, Mohamed Sayyouh, Aine Marie Kelly

**Affiliations:** 1 Department of Radiology, Division of Cardiothoracic Radiology, University of Michigan, 1500 East Medical Center Drive, Ann Arbor, MI 48109 USA; 2 Fulford Radiology, Base Hospital, Private Bag 2016, New Plymouth, Taranaki 4342 New Zealand

**Keywords:** Cardiac disease, Incidental finding, Significant, Chest computed tomography (CT), Management

## Abstract

**Objective:**

Incidental cardiac findings are often found on chest CT studies, some of which may be clinically significant. The objective of this pictorial review is to illustrate and describe the appearances and management of the most frequently encountered significant cardiac findings on non-electrocardiographically gated thoracic CT. Most radiologists will interpret multidetector chest CT and should be aware of the imaging appearances, significance, and the appropriate next management steps, when incidental significant cardiac disease is encountered on thoracic CT.

**Conclusion:**

This article reviews significant incidental cardiac findings which may be encountered on chest CT studies. After completing this review, the reader should not only be familiar with recognizing clinically significant cardiac findings seen on thoracic CT examinations but also have the confidence to direct their further management.

## Teaching points

- With the continued rise in advanced imaging, particularly multidetector row computed tomography (CT), radiologists who interpret trauma or inpatient body CT will diagnose incidental cardiac disease on chest CT, which may require swift action or further management.
- In sick patients and inpatients, thoracic and cardiac comorbidities frequently occur and therefore radiologists should be aware of the appearances of significant cardiac findings on chest CT so that they can direct further appropriate management.

## Introduction

Chest (thoracic and cardiovascular) computed tomography (CT) is commonly performed for a variety of acute and routine clinical indications and is the third most common CT procedure performed after abdominal pelvic and brain CT [1]. The numbers of CT’s performed are also rising, with an average increase of 7.8% annually between 1996 and 2010 [2]. The incidence and prevalence of chest symptoms and disorders in the general adult population is common, and it may be higher in ill in-patients, with many indications generating chest CT examinations. In addition, patients undergoing CT are often middle aged and older and may have existing heart and lung comorbidities, some of which will manifest on thoracic CT. Given that thoracic and cardiac disease etiological factors and disease processes overlap, radiologists who interpret body CT will frequently encounter cardiac findings on chest CT [3].

Most hospitals and outpatient medical centers now utilize multi-detector row CT (MDCT) which allows better spatial resolution and for more efficient throughput and increased numbers of patients scanned [4]. With this increase in CT imaging, body or chest radiologists are encountering more and more incidental findings and the challenge is to determine which findings are significant and will require further action or management. Previous studies of patients undergoing CT (including CT to evaluate for pulmonary embolism) have reported cardiac abnormalities, in 61 to 78% of cases [5, 6]. Similarly, many radiologists will frequently interpret non-cardiac or non-electrocardiographically (ECG) gated thoracic CT and will likely come across cardiac disease and findings, some of which will require further management or specific action to be taken [7–10]. The high volume of thoracic CT examinations without ECG gating (including pulmonary embolism CT) represents an opportunity for radiologists to comprehensively evaluate and comment on the presence or absence of cardiac disease, which may influence future clinical decisions [11].

Some of these cardiac entities will be significant, requiring further management, which may include referral for other imaging, referral to another specialty, intervention, or imaging follow up. Therefore, radiologists should not only be familiar with the appearances of the more commonly occurring significant cardiac findings encountered on thoracic CT but also be able to confidently direct their further management.

Incidental significant cardiac findings that might be encountered on thoracic CT will include shunts (both intracardiac and extracardiac), valvular anomalies and diseases, coronary anomalies and disease, chamber and wall masses, and myocardial and pericardial disease.

The purpose of this pictorial review is to demonstrate the more common and significant cardiac findings seen on non-cardiac, non-ECG gated chest CT (thoracic CT) examinations; discuss their incidence and prevalence when known; describe and illustrate their CT imaging features; generate an appropriate differential diagnosis; and finally, to outline the appropriate next management steps.

### Cardiac shunts

#### Atrial septal defect

About 60% of atrial septal defects (ASDs) are secundum defects, found in the region of the fossa ovalis, and over two-thirds of patients with secundum defects are female [12, 13]. Although, isolated small patent foramen ovale or ostium secundum ASDs have an estimated prevalence of up to 24% in MDCT and coronary CT patients and up to 25–35% in autopsy studies, most are not visualized [14, 15]. Associations include mitral valve prolapse.

Less common ASDs include the ostium primum ASD in the lowermost atrial septum (about 35% of ASDs), associated with Downs syndrome and endocardial cushion defects and presenting early in life [12, 13]. More rare ASDs include the superior sinus venosus defect in the superior atrial septum, usually associated with partial anomalous pulmonary venous return and the inferior sinus venosus defect in the inferior portion of the right atrium at the junction of the inferior vena cava with the right atrium and the coronary sinus ASD, a deficiency of the wall between the coronary sinus and the left atrium [16].

Tiny shunts < 0.5 cm in diameter are usually not of hemodynamic significance with larger shunts > 2 cm being associated with hemodynamic disturbance [17]. Some ASDs will not be diagnosed until the fourth and fifth decades of life and most frequently present with progressive dyspnea on exertion. Atrial septal defects get progressively larger with age due to hypertrophy of the left ventricle with decreased compliance.

**Fig. 1:**
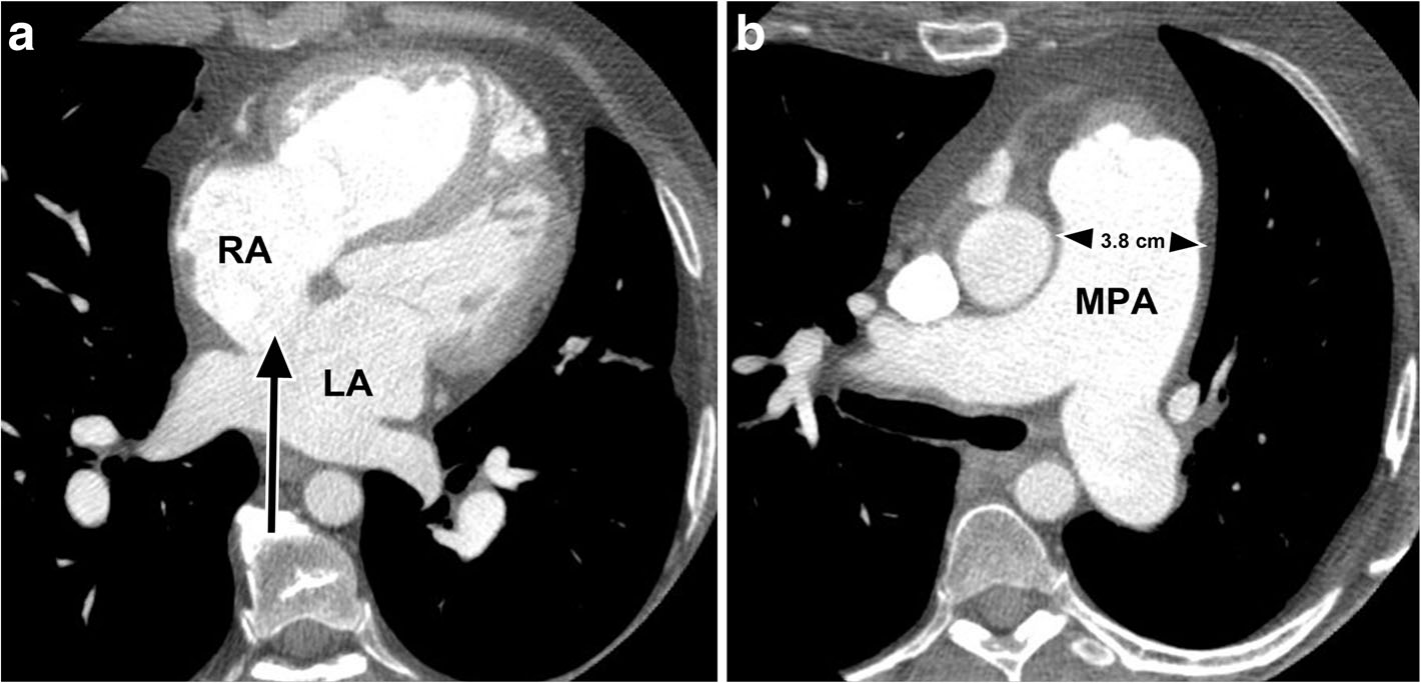
**a** Large ostium secundum ASD (black arrow) in a 51-year old female who was being investigated for cause of pulmonary hypertension. *RA* right atrium, *LA* left atrium. **b** The patient also had large central pulmonary arteries demonstrated by thoracic CT. *MPA* main pulmonary artery

On imaging, larger atrial septal defects may be seen on contrast-enhanced CT as a visible defect in the upper or mid-portion of the atrial septum (Fig. 1). In a pulmonary embolus CT study, contrast-enhanced blood may be visible passing from the right to the left atrium through the defect, but the absence of this does not exclude a shunt. On CT, coronal reformatted images may better demonstrate the ASD, but for more definitive evaluation, suspected ASDs should be further evaluated by echocardiography (ECHO) or magnetic resonance imaging (MRI) to determine the magnitude and direction of flow.

Management involves the use of closure devices, which are nowadays inserted percutaneously, although shunts greater than 3.8 cm (in late systole) are generally not amenable to closure with devices [17]. In general, elective closure is advised for all ASDs with evidence of right ventricular overload or with a clinically significant shunt (pulmonary flow [Qp]-to-systemic flow [Qs] ratio > 1.5) [18]. According to the latest American Heart Association and the British Society for Antimicrobial Chemotherapy guidelines, patients with ASDs no longer require antibiotic prophylaxis before medical and dental procedures [19, 20].

#### Patent ductus arteriosus

The incidence of patent ductus arteriosus (PDA) is estimated to be as high as 1 in 500 and it is twice as common in females [21]. Most cases are sporadic and risk factors include prematurity, with other associations including Rubella infection in early pregnancy, fetal valproate syndrome, and genetic disorders such as Trisomy 21, Holt Oram, and Carpenters Syndrome [21]. Familial cases with an autosomal recessive inheritance have been reported.

There is a wide variability in size and configuration, which determines the degree of shunt and other effects. Patent ductus arteriosus is classified as silent, small, moderate, or large. Some small and moderate-sized PDAs may not present until adulthood, typically after the onset of hypertension, which increases flow through the PDA due to a rise in systemic arterial resistance [17]. Complications in severe cases include pulmonary hypertension and Eisenmengers syndrome. Aneurysms of the ductus arteriosus may also occur as a complication. Associations include ASD and ventricular septal defects (VSD).

**Fig. 2:**
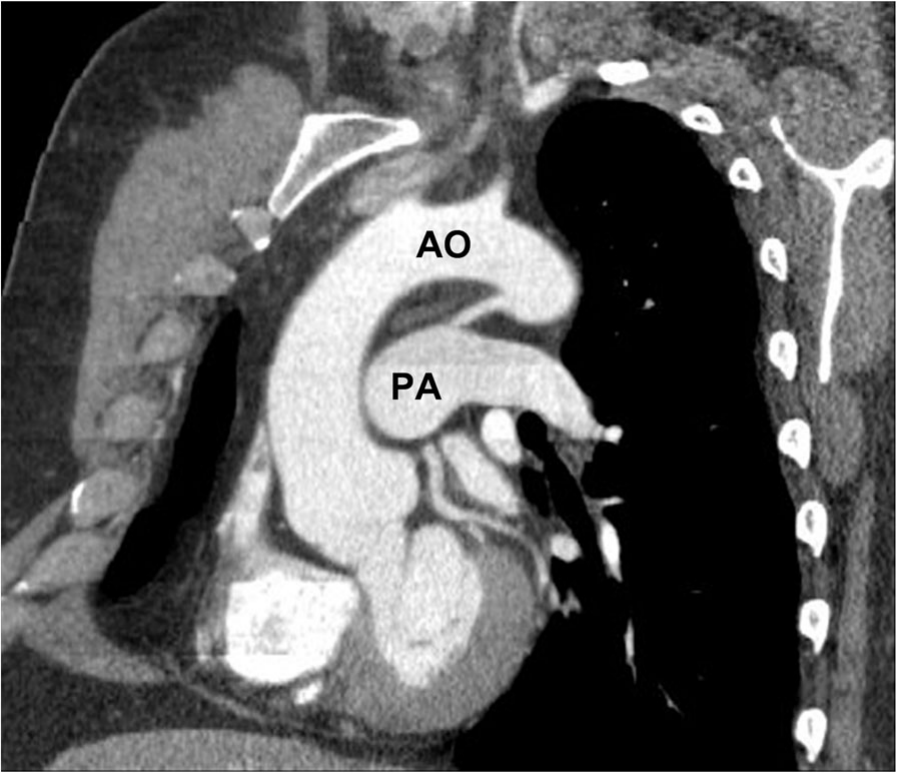
Oblique sagittal reformatted image showing a “tubular” type of PDA, which was an incidental finding in a 52-year male patient being worked up for pulmonary embolism. *AO* aorta, *PA* pulmonary artery. This study was ECG gated and was probably a “double rule out” study to evaluate for pulmonary embolism and Aortic disease

On contrast-enhanced CT imaging, a PDA may be seen as a communication between the undersurface of the aortic arch and the pulmonary artery, often best seen on sagittal or other multiplanar reformatted views, such as double oblique thin section reformatted images on soft tissue windows. Patent ductus arteriosus can be classified into types A (conical, wider at the aortic end and narrower at the pulmonary artery insertion), B (window, large and short and more narrow at the aortic end), C (tubular, narrow with no constrictions) (Fig. 2), D (complex, with multiple constrictions), and E (elongated, with constriction away from the anterior border of trachea) [22].

Echocardiography is recommended as the first step to diagnose and characterize the PDA. Nowadays, closure for symptomatic shunts is indicated unless there is fixed high pulmonary vascular resistance. This is usually performed with transcatheter closure techniques, including coil insertion as well as occluder devices. For asymptomatic shunts, closure is indicated if there is left atrial enlargement resulting from the left-to-right shunting. According to the latest society guidelines, patients with PDA’s no longer require antibiotic prophylaxis before medical and dental procedures [19, 20].

### Valvular disease

#### Sinus of Valsalva aneurysm

Sinus of Valsalva aneurysms (SOVA) are rare with an incidence of about 0.09% of the general population [23]. The exact incidence is unknown, however, as these lesions are frequently silent. They account for 0.1–3.5% of all congenital heart defects and can also be acquired with connective tissue disorders and trauma [24, 25]. In SOVA, there is a weakness in the elastic lamina at the junction of the aortic media and the annulus fibrosus, which in congenital cases, is associated with Marfan’s syndrome, Ehlers-Danlos syndrome, or bicuspid aortic valve. In acquired SOVA, secondary degeneration of the elastic connective tissue can occur due to connective tissue disease, atherosclerosis, infection (e.g., bacterial endocarditis, syphilis, and TB), or trauma (including iatrogenic). There is a 4:1 male predominance and the reported incidence is higher in Asian people.

Sinus of Valsalva aneurysms occur mainly in the right coronary sinus (75–90%), less often in the non-coronary sinus (10–25%), and least often in the left coronary sinus (and are frequently acquired in this location).

The upper limits of normal for sinus diameter in men is 4 cm and in women, 3.5 cm, with slight variations, when adjusted for body surface area [23]. Depending on the location of the aneurysm, there may be compression of other structures by the aneurysm. Aortic regurgitation is a complication. Surgery is indicated for ruptured cases, and for cases with associated aortic regurgitation. Management of un-ruptured SOVA is controversial, but elective intervention is recommended for large lesions, to prevent rupture later. Intervention is also indicated where there is compression of the right ventricular outflow tract, or if the lesion is complicated by arrhythmia or infection.

**Fig. 3:**
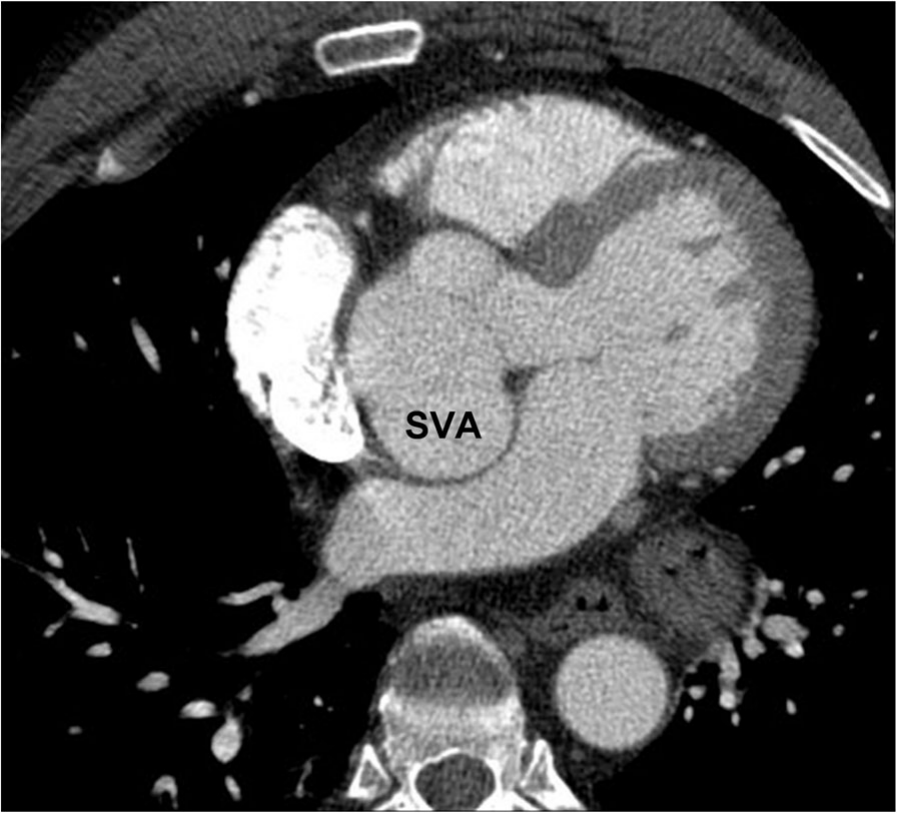
Sinus of Valsalva aneurysm (SOVA) of the non-coronary sinus on an axial contrast enhanced thoracic CT in a 59-year old male being evaluated for thoracic neoplasm. *SVA* sinus of Valsalva aneurysm

On imaging, the distance from the outer edge of the sinus to the opposite aortic wall should not normally exceed 4.5 cm. The aortic sinuses should be evaluated on a double oblique reformatted image at a dedicated 3D workstation, to ensure that the valve ring is completely in the image plane. On imaging, dilatation of the affected aortic sinus is visualized as an enhancing lesion closely related to the aortic root (Fig. 3). Magnetic resonance imaging (MRI) or ECG gated cardiac CT may more easily demonstrate the connection of the lesion to the aortic root and are useful for surgical pre-planning.

#### Bicuspid aortic valve

**Fig. 4:**
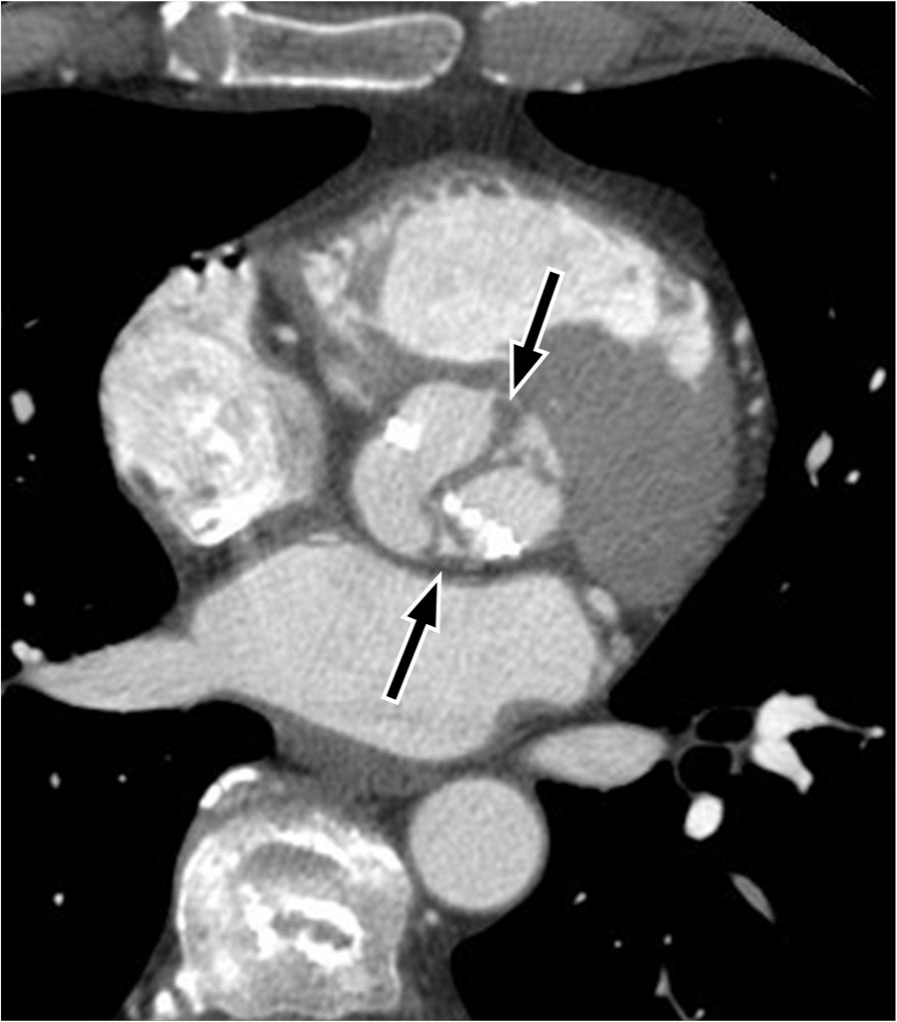
Axial contrast CT shows a bicuspid aortic valve (black arrows) in a 62-year old man being worked up for a widened mediastinum. The valve leaflets are thickened and calcified

Bicuspid aortic valve occurs in 0.5% to 2% of the population and is the most common congenital lesion affecting the human heart [26]. Bicuspid aortic valve (BAV) is an autosomal dominant heritable cause in up to 10% of cases, with variable penetrance. Bicuspid aortic valve is associated with aortic root and ascending aortic dilatation, aortic coarctation (COA), Turner’s syndrome, patent ductus arteriosus, supravalvular aortic stenosis (Williams syndrome), and ventricular septal defect. Most patients with BAV will develop some complications during life, which include aortic stenosis, aortic regurgitation, and infective endocarditis. Bicuspid aortic valve is thought to underlie up to 50% of cases of aortic stenosis [27]. Classification of BAV is based on three characteristics: the number of raphes [fibrous ridges between conjoined cusps] (types: 0, valve with no raphe; 1, valve with one raphe; and 2, valve with two raphes), the spatial position of cusps and raphes (phenotypes: antero-posterior or right-left), and the functional status of the valve [28]. The anterior-posterior phenotype with raphe (Fig. 4) is the most common and is associated with COA.

Anatomically tricuspid but functionally bicuspid valves (secondary bicuspid valves) are more common than congenital bicuspid valves and can be difficult to distinguish from each other on imaging. Double oblique reformatted images (on a dedicated 3D workstation) can be viewed to see the valve in plane, but for definitive differentiation and evaluation for the need for operative intervention, functional information will be required, which can be obtained using ECHO or MRI.

On imaging, the cusps of the bicuspid valve are usually of unequal size (as one of them is two cusps fused with or without a raphe). Calcification may be seen at the base of the cusps or at the raphe. Ascending aortic dilatation occurs mostly due to cystic medial necrosis (bicuspid aortopathy) rather than secondary to valve stenosis (post stenotic dilatation) (Fig. 4). Patients with BAV and aortic root or ascending aortic diameters greater than 4 cm should undergo serial evaluation with MRI or CT, and yearly evaluation when the aortic diameter exceeds 4.5 cm [29]. Operative intervention is recommended when the aortic root or ascending aortic diameter is greater than 5.5 cm or if the rate of increase is more than 0.5 cm per year [29, 30].

### Coronary arterial disorders

#### Coronary artery disease

Coronary arterial calcification (CAC) is a marker of the burden of coronary artery atherosclerosis but its relationship to plaque instability is less predictable. Coronary arterial calcification can be an early indicator of coronary artery disease in asymptomatic individuals being worked up for surgery or other suspected chest pathology. Coronary artery disease was the most frequent cardiac finding on chest CT in prior studies with the left anterior descending coronary artery being the most frequently involved [6]. The same authors note that the presence of coronary arterial calcification was often not mentioned in chest CT reports [6]. Coronary arterial calcification can be easily seen even on non-ECG gated unenhanced CT. Radiologists interpreting chest CT should not only evaluate the anatomic origin of the coronary arteries but also evaluate for the extent and severity of coronary arterial disease. Patients with coronary arterial calcification have increased risk for cardiac events, including ischemia, arrhythmia, hypotension, myocardial infarction, coronary arterial intervention or surgery, and death. For each standard deviation increase in CAC scores on non-ECG gated CT, the odds ratio for death increased by 50%, when adjusted for traditional cardiovascular disease risk factors [31]. Detection of coronary arterial calcification might enable the patient to undergo primary preventative measures such as dietary changes, exercise, aspirin, or statin treatment. Coronary arterial calcification has a high specificity and negative predictive value [6]. In patients who are undergoing CT prior to surgery, it is important for the surgeon and anesthetist to know about the coronary arterial calcification burden, as the patient may need functional imaging or cardiology consultation first or a different surgical approach or anesthetic plan.

#### Coronary artery ectasia and aneurysm

Coronary artery aneurysms are commonly defined as a 50% or greater localized increase in diameter of the affected vessel compared with the adjacent arterial segment [32]. Giant coronary artery aneurysms can reach several cm in diameter [33]. Coronary artery ectasia is defined as diffuse coronary artery dilation with less than 50% increase in diameter [34]. Coronary artery aneurysms are most commonly caused by atherosclerosis in adults or by Kawasaki disease in children [35]. Other etiologies include trauma (including iatrogenic), arteritis (granulomatosis with polyangitis, polyarteritis nodosa, systemic lupus arteriosus), infection (syphilis, staphylococcus), dissection, connective tissue disorders (Marfan’s, Ehlers-Danlos), and cocaine abuse [35].

**Fig. 5:**
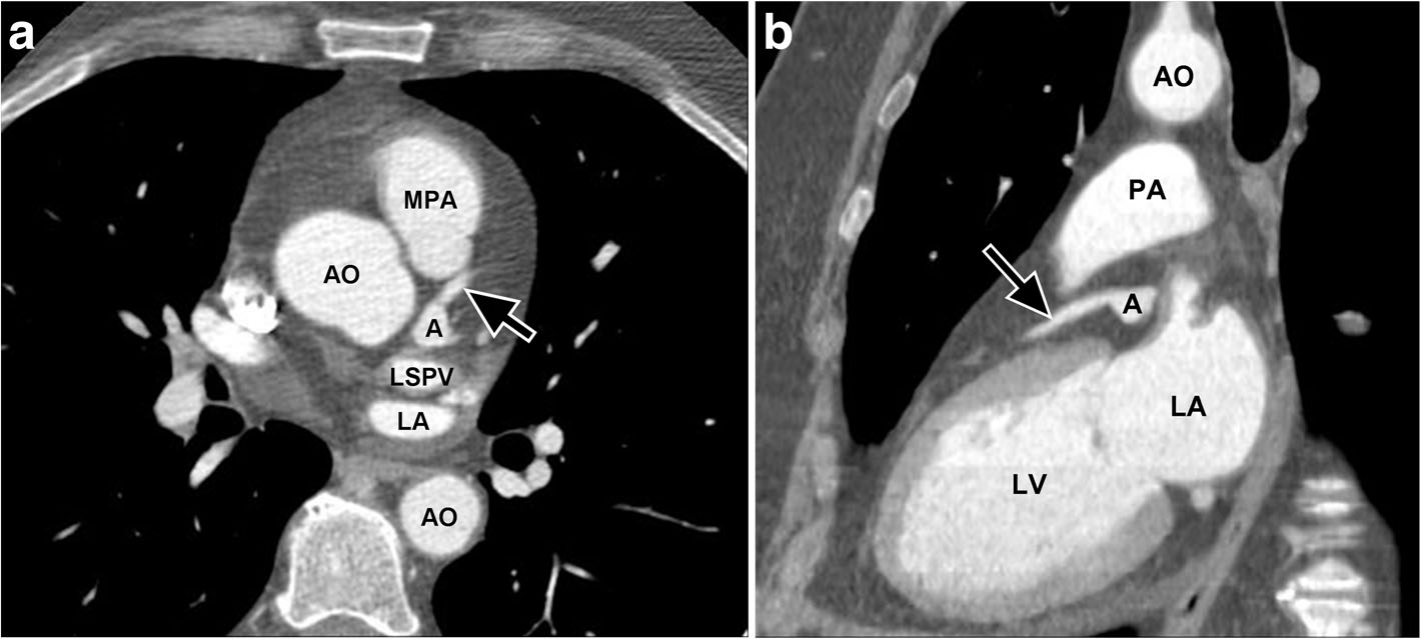
**a** Axial contrast enhanced CT image demonstrating a coronary artery aneurysm (**a**) of the left anterior descending coronary artery (black arrow) in a 56-year-old female who was being evaluated for aortic disease. **b** Multiplanar reformatted images in the sagittal oblique plane also demonstrate the aneurysm (**a**) of the left anterior descending coronary artery (black arrow). *A* aneurysm, *AO* aorta, *PA* pulmonary artery, *LA* left atrium, *LSPV* left superior pulmonary vein. Please note that this examination was an ECG gated aortic CTA, but images were included as it was such a good example of coronary artery aneurysm

Patients may be asymptomatic or present with heart failure or angina due to embolic or thrombotic phenomena. Hypertension is a risk factor. Aneurysms are most commonly found in the right coronary artery (RCA, 40%), followed by the left anterior descending (LAD, 32%) and circumflex (LCX, 23%) coronary artery [36]. Aneurysms have traditionally been diagnosed by echocardiography (ECHO) or catheter angiography, but the latter may underestimate the size of the aneurysm if there is considerable intraluminal thrombus [37]. Nowadays, they are increasingly found on CT and MR imaging [35]. On imaging, coronary artery aneurysms are seen as fusiform dilatations of a portion of the coronary artery (Fig. 5). Some have classified them according to the extent of involvement (diffuse or discrete ectasia and number of vessels affected). Differential diagnosis includes coronary artery fistula and aberrant origin of the coronary artery from the pulmonary artery (Bland-White-Garland syndrome) [34].

Further evaluation can be carried out non-invasively with electrocardiographically (ECG) gated cardiac CT or cardiac magnetic resonance imaging (CMR). Management is unclear with some advocating surgical or endovascular repair (usually to treat any areas of stenosis in cases of atherosclerosis) and others taking a more conservative approach, including anti platelet agents and anticoagulation [38].

#### Coronary artery fistula

A coronary artery fistula (CAF) is defined as a single or multiple direct precapillary connections between a branch of a coronary artery and the lumen of a cardiac chamber (coronary cameral fistula), or an arterial/venous structure including the coronary sinus, superior vena cava, pulmonary artery, pulmonary vein, or bronchial vein (coronary arteriovenous fistula) [39, 40]. Coronary artery fistulas are rare, representing 0.2 to 0.4% of all congenital heart disease (CHD), 14% of all coronary anomalies, and often incidentally found on angiographic imaging [41, 42]. The majority of CAF are congenital, but some are acquired secondary to trauma, infection, neoplasms, or iatrogenic injury. They can also occur as a result of intracardiac congenital heart operations, transcutaneous techniques used for myocardial biopsy or coronary angioplasty, or as a complication of Kawasaki disease [41, 43]. As more cardiac transplants are performed and patients undergo myocardial biopsies, the incidence of acquired CAF in this patient population is increasing.

Fistulas most commonly arise from the RCA (50 to 60% of cases), the left anterior descending coronary artery (25 to 42%), the circumflex (18%), or both coronary arteries (5%) [33, 45]. Single fistulas are more common (74 to 90%) than multiple (10 to 16%) [43]. The most common (60 to 90%) site of drainage is the right side of the heart [44–46]. The remaining 10% may be connected to the pulmonary artery, coronary sinus, superior vena cava (SVC), pulmonary vein, left atrium (LA), left ventricle (LV), or have multiple connections [47]. Termination into a cardiac chamber or vascular structure with lower pressure may lead to enlargement and tortuosity of the artery [46]. The clinical presentation of CAFs depends on the severity of the left-to-right shunt, with the majority detected in adult patients being usually asymptomatic. Coronary angiography can reliably demonstrate the proximal part of the CAF and allow evaluation of the size and number of fistulas present. However, coronary fistulas draining into low-pressure chambers of the heart may not be well-visualized by conventional angiography because of significant dilution of the contrast medium. Electrocardiographically gated MDCT is useful to image CAF and provide a road map for treatment planning [39].

**Fig. 6:**
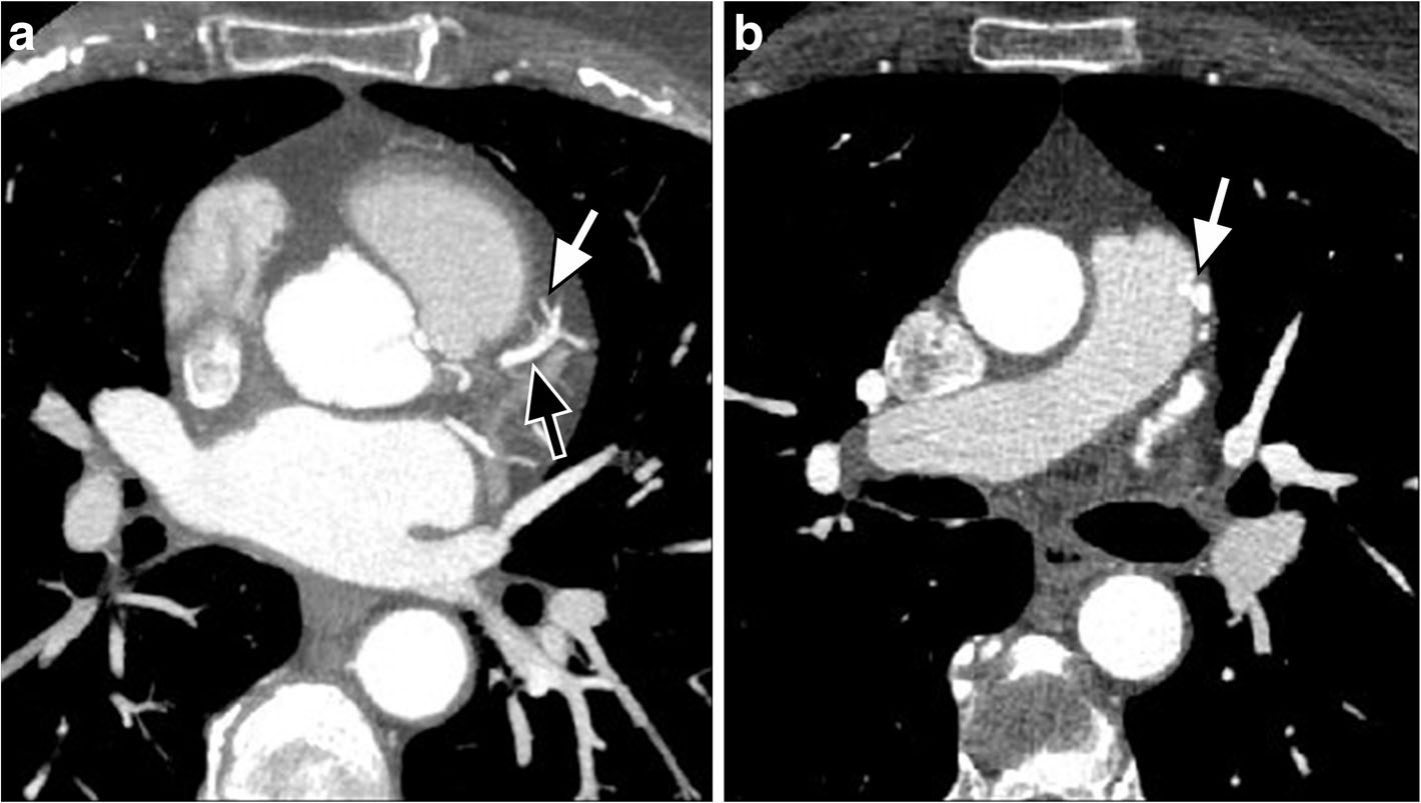
**a**, **b** A 62-year-old woman being evaluated for weight loss. **a** Axial thick slab maximum intensity projection (MIP) CT image shows the coronary artery fistula (white arrow) arising from the left anterior descending coronary artery (black arrow). **b** Axial CT image shows the coronary artery fistula emptying into the main pulmonary artery (white arrow). Please note that this examination was an ECG gated aortic CTA, but images were included as it was such a good example of coronary artery fistula

Symptomatic CAF (most commonly dyspnea due to pulmonary hypertension or heart failure as a result of left to right shunting, chest pain due to chronic angina secondary to a coronary “steal” phenomenon) were usually treated with surgery, though nowadays some can be treated percutaneously [40, 47]. Management of asymptomatic CAF is more controversial, with some advocating surgical management for larger fistulae (fistula luminal diameter is ≥ 2, the luminal diameter of the reference vessel) [40, 45]. On CT imaging, single or multiple connections will be seen passing from the coronary artery to a cardiac chamber, pulmonary or systemic artery (Fig. 6). Referral to a cardiologist for further evaluation (which may include ECG gated cardiac CT or MRI for surgical planning with unclear etiology) is recommended.

#### Anomalous coronary artery origin

Coronary arteries can have anomalous origins from a contralateral sinus of Valsalva, the pulmonary artery, or another coronary artery or its branches [48–50]. The most serious anomaly occurs when a left or right coronary artery arises from the contralateral sinus of Valsalva and courses between the aorta and pulmonary trunk (intra-arterial or “malignant” course). Other high-risk anatomic features include a slit-like orifice, an intramural course (through the aortic wall), and an acute take off angle [51]. Death is thought to be caused by ischemia resulting from ostial occlusion due to angulation or kinking at the coronary orifice or compression within the aortic wall or between the aorta and the pulmonary artery during diastole [46].

**Fig. 7:**
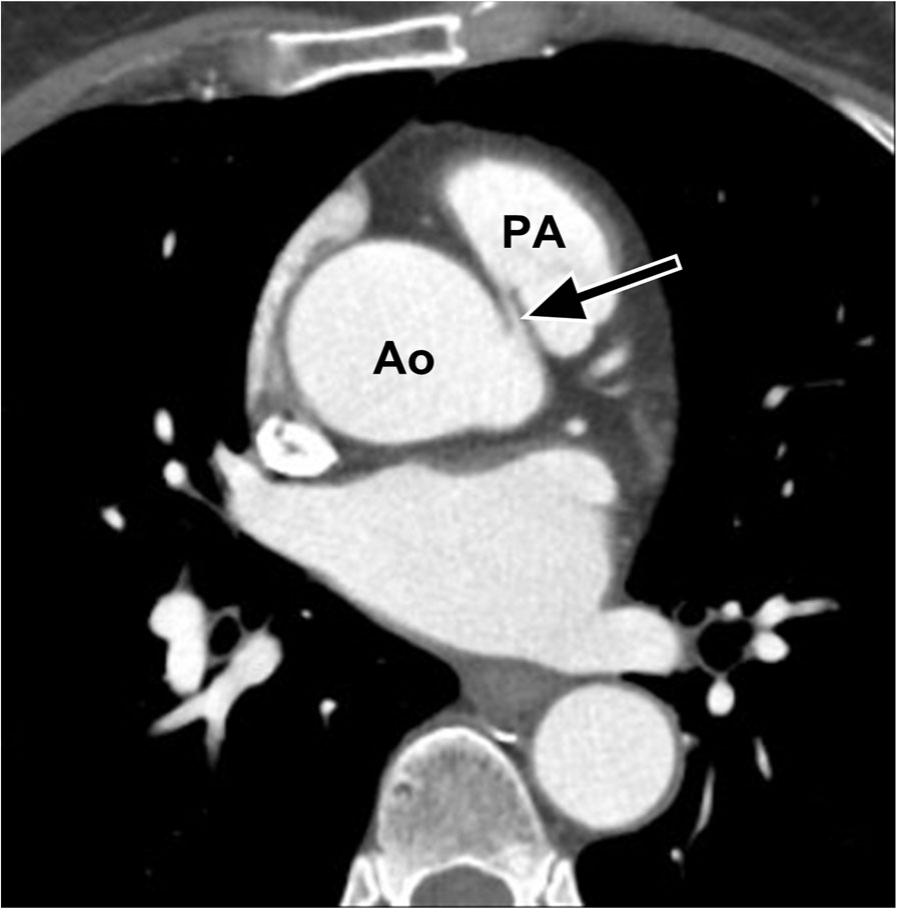
Axial contrast enhanced CT demonstrates the RCA (black arrow) arising from the left coronary cusp in a 43-year-old female being evaluated for hoarseness and vocal cord paralysis. *Ao* aorta, *PA* pulmonary artery

In contrast, anomalous coronary arteries that course anterior to the pulmonary artery (pre-pulmonic), posterior to the aorta (retro-aortic), or more caudal (to the pulmonary valve) across the ventricular septum (trans-septal) do not usually have hemodynamic consequences and are regarded as “benign.” An origin of the left coronary artery from the pulmonary artery (Bland-White-Garland syndrome) or less commonly origin of the right coronary artery from the pulmonary artery is also considered as malignant as it is frequently associated with myocardial ischemia and death in early childhood [46]. Electrocardiographically gated MDCT coronary angiography has emerged as the standard of reference for evaluation of coronary artery anomalies. On imaging, the abnormal origin and course will be visible as a contrast-enhanced vessel passing anterior to the pulmonary artery, posterior to the aorta, or coursing between the two vessels (Fig. 7). Sagittal reformatted images are useful for evaluating whether the course is intra-arterial or trans-septal, which has implications for treatment [52]. Treatment of “malignant” anomalous coronary arteries is usually surgical, but there have been a few reports of successful endovascular repair in cases with functional obstruction [53, 54].

### Abnormalities of the cardiac walls

#### Left ventricular aneurysm, pseudoaneurysm, and diverticulum

It can be difficult to distinguish true left ventricular aneurysms from pseudoaneurysms or congenital diverticula, but correct differentiation is crucial since pseudoaneurysms have a high risk of rupture and surgical repair is recommended, while true aneurysms and diverticula can often be managed medically. Two features which may help distinguish these entities include location and ostial (neck) diameter.

**Fig. 8:**
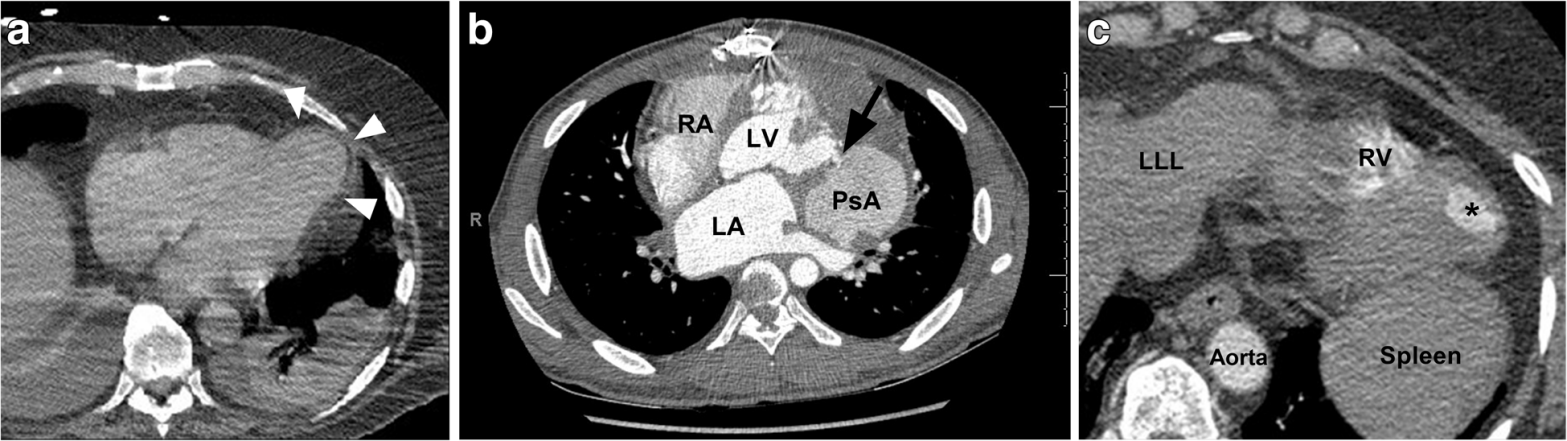
**a** Axial contrast-enhanced CT demonstrates a left ventricular true aneurysm (white arrowheads) in a 71-year-old male who was being evaluated for empyema. He had a history of myocardial infarction. **b** Axial CT image through the chest demonstrates a left ventricular pseudoaneurysm with a narrow neck (black arrow) in a young male being evaluated for fever and chest pain. *LA* left atrium, *RA* right atrium, *LV* left ventricle, *PsA* pseudoaneurysm. **c** Axial CT image through the lower chest and upper abdomen demonstrates a small apical left ventricular diverticulum (asterisk) in a 55-year-old male being evaluated for thoracic neoplasm. *LLL* left liver lobe, *RV* right ventricle

Left ventricular (LV) true aneurysms occur after myocardial infarction with the majority at the apex, or anterolateral wall (Fig. 8) [55]. More posterior infarcts are thought to be lethal due to involvement of the papillary muscles and associated severe mitral valve regurgitation, with less reported. Left ventricular true aneurysms are thinned areas of scarred myocardium which can be dyskenitic or akinetic, and complicated by heart failure, arrhythmia, stasis of blood flow, thrombus, and emboli.

Pseudoaneurysms are usually located posteroinferiorly and may be caused by ischemia, infarction, or trauma (Fig. 8) [55]. A pseudoaneurysm occurs when rupture of the free LV wall in myocardial infarction is contained by overlying adherent pericardium or not contained. Left ventricular pseudoanerysms usually rupture, resulting in immediate death, and as a result are not commonly seen by imaging. If the patient survives, pseudoaneurysms may cause congestive heart failure with embolic events as the cavity is noncontractile or dyskinetic, due to slow flow of blood and thrombosis [55]. Left ventricular aneurysms tend to have wide, more evident openings in contrast to the narrow ostium of pseudoaneurysms or diverticula which can be difficult to visualize [55, 56].

Diverticula are rare congenital anomalies and typically include endocardium, full thickness myocardium and pericardium, arising near the apex, except for the isolated fibrous variety, found at the heart base or in the subvalvular area (Fig. 8) [56, 57]. Muscular diverticula are often associated with midline defects involving the abdominal wall (omphalocoele), sternum, diaphragm, and heart (ASD) (Cantrell syndrome) [58]. Left heart catheterization (ventriculography) was the gold standard to visualize muscular diverticula contracting in systole, in contrast to pseudoaneurysms which do not contract. Nowadays, dynamic imaging with ECG gated MDCT or functional cine MRI can demonstrate the form and function less invasively. The presence of myocardium surrounding the aneurysmal cavity suggests a true aneurysm (thinned myocardium) or diverticulum (full thickness myocardium) and myocardial discontinuity suggests pseudoaneurysm.

Finally, true aneurysms in any location within the left ventricle are much more common than pseudoaneurysms or diverticula; therefore, location is not an adequate criterion for clinical decision making. The more posterior the outpouching, the more difficult it is to detect [59].

### Myocardial disease

#### Dilated cardiomyopathy

**Fig. 9:**
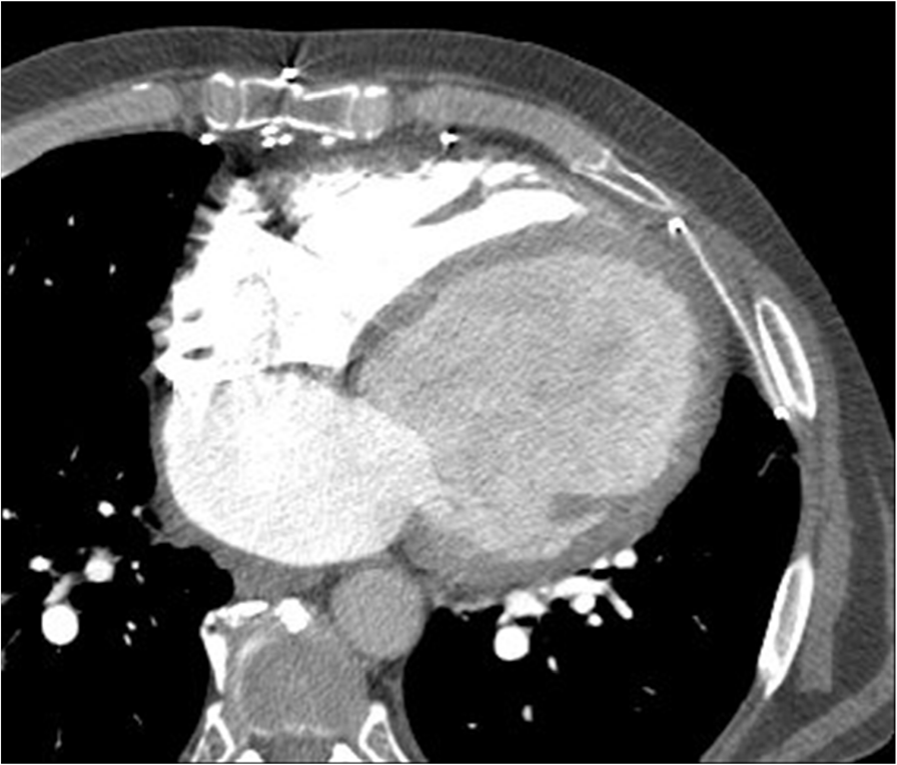
Axial contrast-enhanced CT demonstrates dilated cardiomyopathy (DCM) on an axial contrast enhanced image in a 65-year-old man being evaluated for PE

In dilated cardiomyopathy (DCM), there is impaired contraction of the left or both ventricles in the absence of ischemic heart disease [60]. Its etiology is unknown in about half of cases (idiopathic DCM) but may be genetic, viral, metabolic, or toxic. Patients present clinically with progressive heart failure. Complications include arrhythmia, thromboembolism, or sudden death [61]. Ejection fraction is reduced and end systolic and end diastolic left ventricular volumes are increased. Diagnosis may be made on ECHO but results are variable, and nowadays MRI is the modality of choice for diagnosis and follow up [62]. On axial non-ECG gated CT images, if a dilated left ventricular chamber is seen (greater than 5.5 cm) with a uniform left ventricular wall thickness less than 0.7 cm, further evaluation with MRI and or ECHO should be suggested (Fig. 9). This is because the left ventricular wall will be thinner (and the chamber more dilated) in end diastole.

#### Left ventricular hypertrophy

**Fig. 10:**
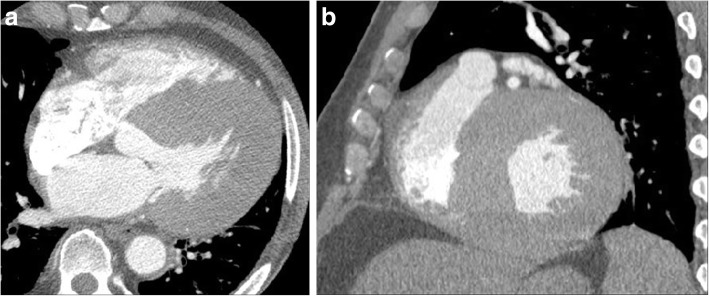
**a** Axial contrast-enhanced CT demonstrates left ventricular hypertrophy (LVH) on an axial contrast enhanced image in a 70-year-old man being evaluated for back pain. **b** Sagittal oblique multiplanar reformatted MPR images (short axis) demonstrate concentric hypertrophy of the left ventricular wall

Left ventricular hypertrophy (LVH) is assessed using estimates of left ventricular mass, which usually includes measurements of end diastolic left ventricular mid cavity diameter, left ventricular posterior wall, and septal wall thicknesses. On non-gated CT, left ventricular wall thickness is likely to be overestimated since imaging may not represent end diastole. However, some suggest LVH when the free or septal ventricular wall exceeds 2.0 to 2.5 cm in thickness on axial CT [3]. If LVH is suspected on CT, a search should be made for etiologies including aortic or mitral valve disease, coarctation, or a left to right shunt (Fig. 10).

LVH is usually diagnosed by electrocardiogram or on echocardiography (ECHO) and if suspected on non-ECG gated CT, further evaluation should be recommended using these modalities and by cardiac MRI [63].

#### Hypertrophic cardiomyopathy

**Fig. 11:**
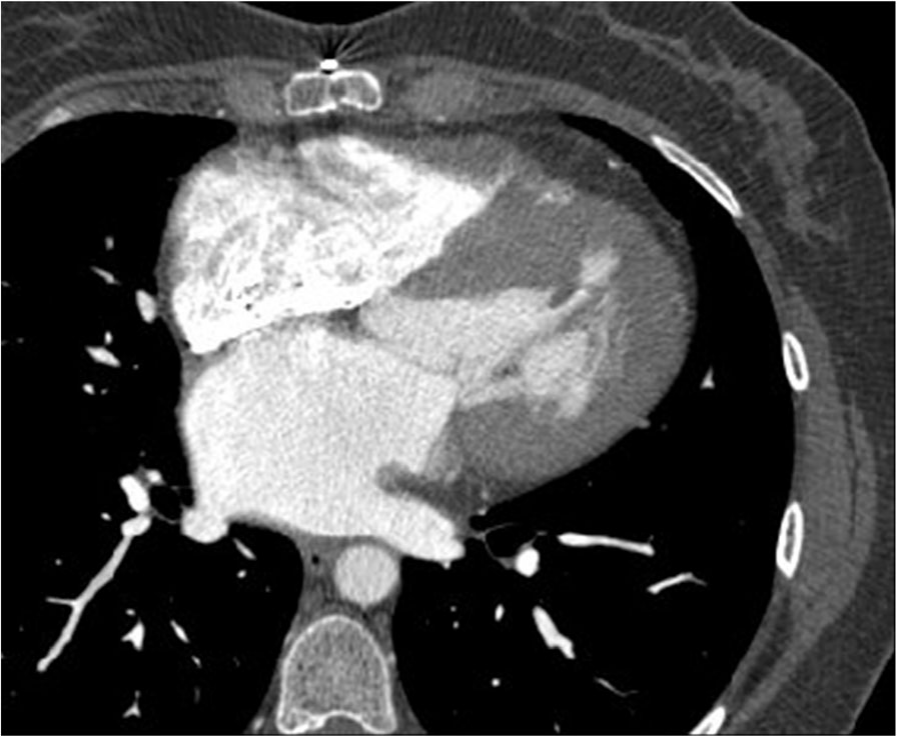
Axial contrast enhanced CT demonstrates the asymmetric form of hypertrophic cardiomyopathy (HCM) on an axial contrast enhanced image (with anteroseptal or mid ventricular thickening) and anatomic narrowing of the left ventricular outflow tract (LVOT) in a 37-year-old female being evaluated for pulmonary embolus

Hypertrophic cardiomyopathy (HCM) is a genetic disorder, with an incidence of 1 in 500, typically with autosomal dominant inheritance, variable penetrance, and expression [60]. Myocardial hypertrophy occurs in the absence of any hypertrophic stimulus and HCM is the most common cardiac cause of sudden death in apparently healthy young adults and athletes. Diagnosis is usually made by ECHO and other testing including ECG and confirmed by cardiac catheterization or MRI imaging which will demonstrate a hypertrophied non-dilated left ventricle. In HCM, myocardial hypertrophy is typically heterogenous, with several forms including asymmetric hypertrophy (the most common, with anteroseptal or mid ventricular thickening) (Fig. 11), symmetric hypertrophy, apical hypertrophy, and mass like hypertrophy [64, 65].

Asymmetric septal wall hypertrophy causes obstruction of the left ventricular outflow tract (LVOT) in up to a third of cases. The mitral valve may be affected secondary to left ventricular outflow tract obstruction with systolic anterior wall motion (SAM), or because of a primary abnormality in the valve itself. Anatomic narrowing of the LVOT during systole and SAM contribute to cause dynamic subaortic obstruction [64, 65]. On axial CT images, narrowing may be seen at the level of the left ventricular outflow tract, in association with thickening of the mid ventricular septum, and non-invasive assessment of the anatomic and functional changes with MRI can be recommended [60, 64, 65]. In older patients, a sigmoid shape to the interventricular septum may be seen but is not usually associated with hemodynamic changes [66].

#### Myocardial calcification

**Fig. 12:**
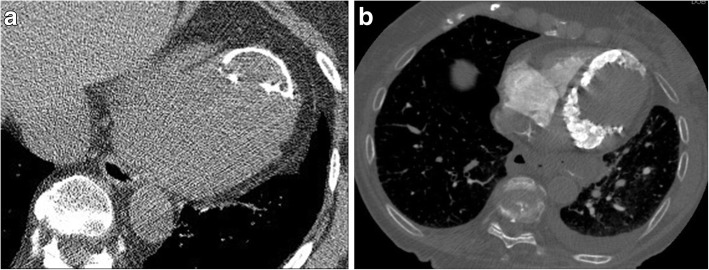
**a**, **b** Axial (**a**) and sagittal non-contrast CT image demonstrating curvilinear dystrophic calcification at the left ventricular apex in a 64-year-old female who was being evaluated for COPD. She had a history of previous myocardial infarct. **b** Axial non-contrast CT on bone window setting demonstrates metastatic myocardial calcification in the left ventricle in an 88-year-old woman with chronic renal impairment

Myocardial calcification may be dystrophic, metastatic, or idiopathic [67, 68]. Dystrophic calcifications are more common and most often seen following myocardial infarction, with calcification reported in 8% of cases 6 years after infarction, and the most common site is in the anterior wall of the left ventricle (LV) [67]. The myocardial calcification results from necrosis, hemorrhage, or fibrosis and usually underestimates the size of the infarct. On CT imaging, dystrophic myocardial calcification is thin and curvilinear and usually found within the periphery of the infarct, at the interventricular septum and apex, distributed away from the aortic root (Fig. 12). Apical left ventricular aneurysms commonly calcify. Other causes of myocardial calcification include trauma, infection, inflammation, and neoplasm [68].

Metastatic calcification is associated with elevated serum calcium and is most commonly reported in chronic renal failure patients on hemodialysis [68]. Globular, diffuse, amorphous, or patchy metastatic calcifications are found in many locations including the ventricular walls (Fig. 12). Metastatic calcification can also be seen with primary, secondary, and tertiary hyperparathyroidism.

The prevalence, etiology, and mechanisms of idiopathic myocardial calcifications are unknown, but some believe these to be dystrophic or metastatic, and secondary to a clinically occult myocardial abnormality [68].

#### Left ventricular myocardial fat

**Fig. 13:**
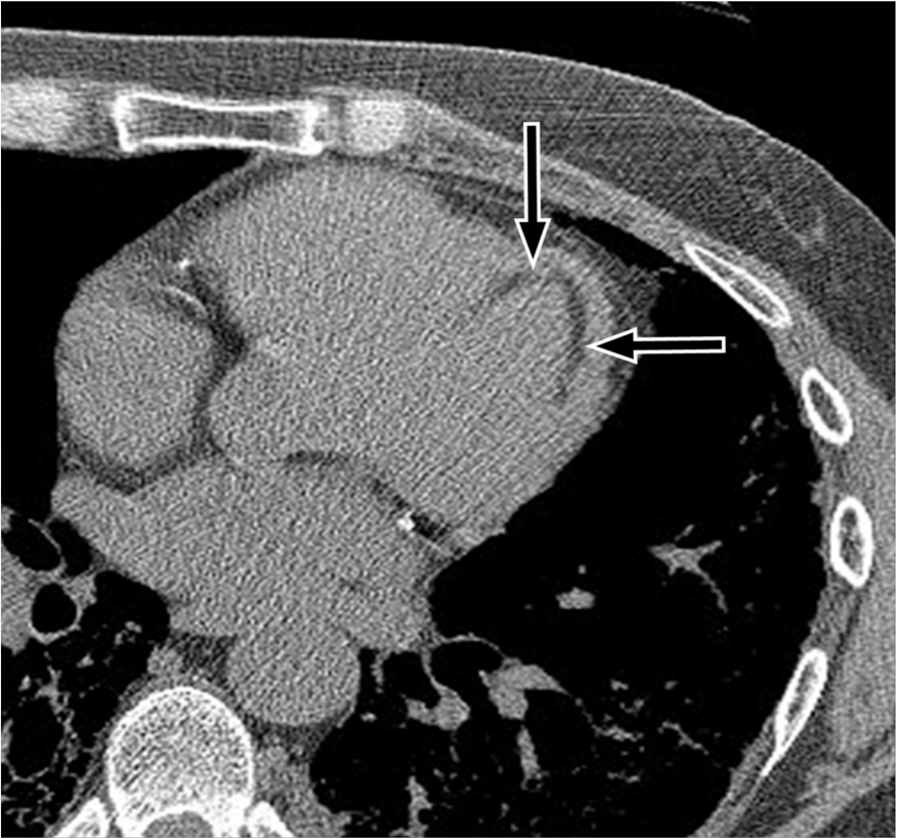
Axial image demonstrates fat replacement in an apical left ventricular infarct on a patient being evaluated for chronic dyspnea by high resolution computed tomography (HRCT)

Fat replacement of the myocardium has been described in up to 60% of patients with chronic left ventricular myocardial infarction [69–71]. The fat is typically thin and linear and follows the distribution of a coronary artery territory, most commonly within a subendocardial location, often in association with left ventricular wall thinning or calcification (Fig. 13). An association with severe heart failure has been described. Myocardial fat is also seen with lipomatous hypertrophy of the interventricular septum, cardiac lipoma, liposarcoma, tuberose sclerosis, and other cardiomyopathies such as HCM and DCM [69–71].

#### Right ventricular myocardial fat

**Fig. 14:**
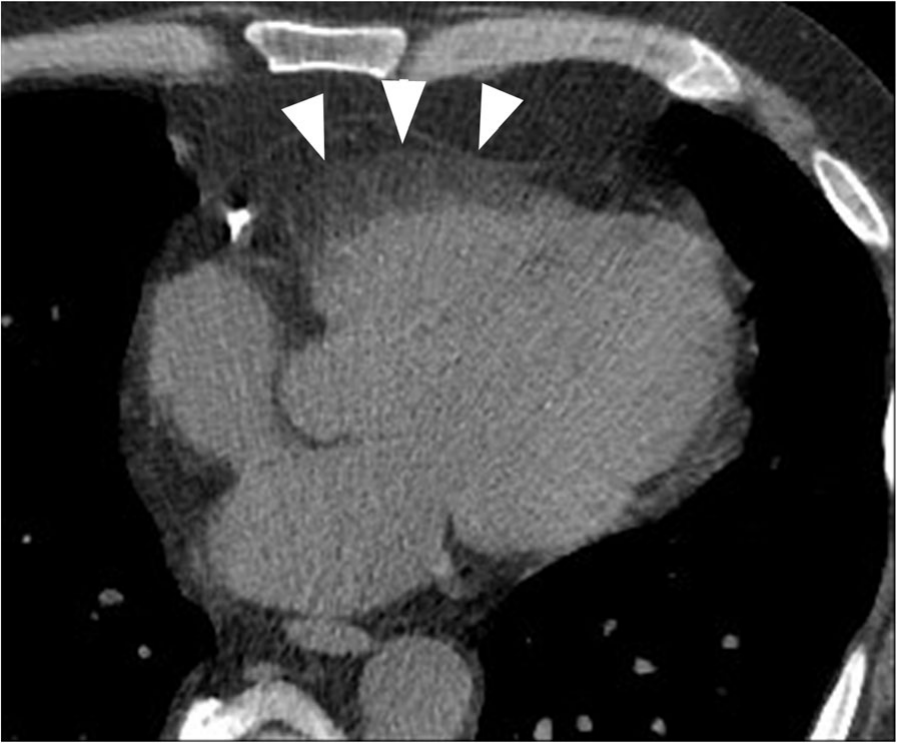
Axial non-contrast CT (which was performed to follow up a lung nodule) demonstrates fat replacement in the free wall of the right ventricle (white arrowheads) in a 50-year-old male

Fat interspersed in the right ventricular myocardium has been described in up to 11% of screening populations and in more than half of normal hearts at autopsy and has been shown to be significantly age related [69–72]. This is more commonly seen in women and is usually of no consequence. A strong correlation with excess body fat has not been proven. An important differential diagnosis for right ventricular fat is arrhythmogenic right ventricular cardiomyopathy/dysplasia (ARVC/D), or a myocardial infarct. These latter two entities are much less common than age-related right ventricular fat. Age-related right ventricular myocardial fat is found in the anterolateral ventricular free wall and right ventricular outflow tract (RVOT), whereas ARVC/D involves the inferior wall, anteroapical wall, and the RVOT (Fig. 14).

Finding right ventricular myocardial fat on CT is only one of many diagnostic features of ARVC/D, and CT has not been included in the diagnosis task force criteria used in the of ARVC/D, but there may be a role for dynamic, 4-D cine cardiac CT in the future [73]. Arrhythmogenic right ventricular dysplasia also occurs in a younger (and symptomatic) patient population and there is often associated right ventricular wall dilatation [74]. The diagnosis of ARVC/D is challenging, due to the absence of unique diagnostic criteria, incomplete penetrance, and variable disease expression. In addition, diagnostic tests are not sensitive enough, findings are non-specific, and the diagnostic criteria used are subjective [73]. Therefore, if ARVC/D is suspected, referral to a cardiologist for complete assessment of diagnostic task force criteria for ARVC/D (which include features on cardiac MRI) is recommended.

### Chamber enlargement

#### Left atrial enlargement

Left atrial enlargement can be caused by mitral valvular disease (stenosis and regurgitation), atrial fibrillation, left to right shunts, left ventricular hypertrophy (LVH), left ventricular failure, dilated cardiomyopathy, and ischemic heart disease. A search should be made for thrombus in the left atrium or its appendage. In addition, evaluation of the mitral valve for calcification or thickening can be made, as well as checking the other chamber sizes and wall thicknesses, and looking for coronary arterial calcification. Left atrial anteroposterior measurements should ideally be made on a three-chamber view (aortic outflow tract, left atrium, and left ventricle) at the end of left ventricular systole [75]. Measurements of the largest anteroposterior left atrial diameter (made at the level of the aortic valve), greater than 4.5 cm on axial non-ECG gated CT, had 94% specificity (53% sensitivity) for left atrial dilatation [76]. Normalized reference range volume measurements for the left atrium and other cardiac chambers have been established using dynamic magnetic resonance imaging by Maceira and colleagues [77].

#### Right atrial enlargement

Right atrial enlargement can be caused by pulmonary arterial hypertension, tricuspid valvular disease (stenosis and regurgitation), atrial fibrillation, left to right shunts, left ventricular hypertrophy (LVH), and ischemic heart disease. There are no accepted standardized measurements for right atrial sizes. Normalized reference range volume measurements for the right atrium and other cardiac chambers have been established using dynamic magnetic resonance imaging [77].

#### Left ventricular enlargement

Left ventricular enlargement (LVE) can be indicative of many pathological entities, including cardiomyopathies, ischemic heart disease and valvular dysfunction.

Non-ECG gated CT images likely will not be obtained in end-diastole and so the ventricular cavity size may be underestimated. A recent study suggested that left ventricular chamber measurements greater than 5.6 cm (made half way between the apex and the valve, from inner wall to inner wall) on axial non-ECG gated CT had a 100% specificity (78% sensitivity) and negative predictive value of 93% (positive predictive value of 80%) [78]. Therefore, dimensional thresholds are best used to provide specific detection (rule in) and to suggest for chamber enlargement, rather than being able to exclude it. Normalized reference range volume measurements for the left ventricle and other cardiac chambers are well established by dynamic magnetic resonance imaging [79].

#### Right ventricular enlargement

Right ventricular enlargement is difficult to assess, due to its complex shape and variability and lack of specific landmarks that can be used as reference points. Right ventricular enlargement is found with pulmonary hypertension, pulmonary valve stenosis or regurgitation, left to right shunts (ventricular septal defect), and cardiomyopathy. Normalized reference range volume measurements for the right ventricle and other cardiac chambers have been established using dynamic magnetic resonance imaging [80].

### Pericardial disease

#### Constrictive pericarditis

**Fig. 15:**
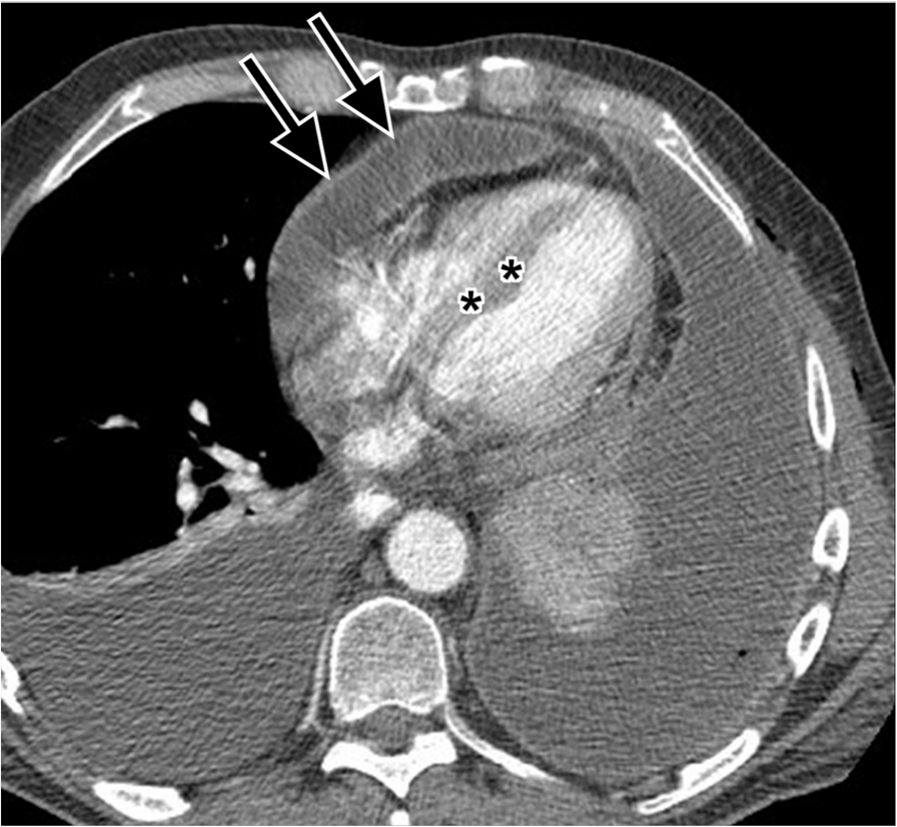
Axial contrast-enhanced CT shows thickening of the visceral and parietal pericardium (black arrows) with fluid in between the pericardial layers in a 77-year-old male patient being investigated for loculated pleural effusion. This pericardial thickening and fluid are resulting in constrictive physiology (constrictive pericarditis), evidenced by a decreased volume, cone-shaped right ventricle, and a sigmoid-shaped interventricular septum (black asterisks)

Constrictive pericarditis results from infections, tuberculosis, connective tissue disorder, neoplasm, or trauma [81]. Although pericardial calcification is found in up to 28% of cases of constrictive pericarditis, the finding does not necessarily cause constrictive physiology [64]. Pericardial constriction from pericardial fluid or from progressive pericardial fibrosis (with a stiff or less compliant pericardium) causes impaired ventricular filling during diastole and diastolic heart failure. This leads to dilatation of the inferior vena cava, hepatic veins, and right atrium with a normal or reduced volume right ventricle (RV). An elongated cone-shaped RV and a sigmoid-shaped interventricular septum are sometimes seen (Fig. 15). Constrictive pericarditis can be difficult to distinguish from restrictive cardiomyopathy clinically and radiologically. However, differentiation is important as constrictive pericarditis can sometimes be treated surgically by pericardiectomy [82]. The two entities can be distinguished on imaging based on whether the pericardium is of normal thickness (restrictive cardiomyopathy) or of abnormally increased thickness (constrictive pericarditis). MR imaging with flow sensitive sequences can help distinguish these two entities [82]. If pericardial constriction is suspected, further imaging with ECHO and cardiac MRI and referral to cardiology is recommended.

#### Hemopericardium

**Fig. 16:**
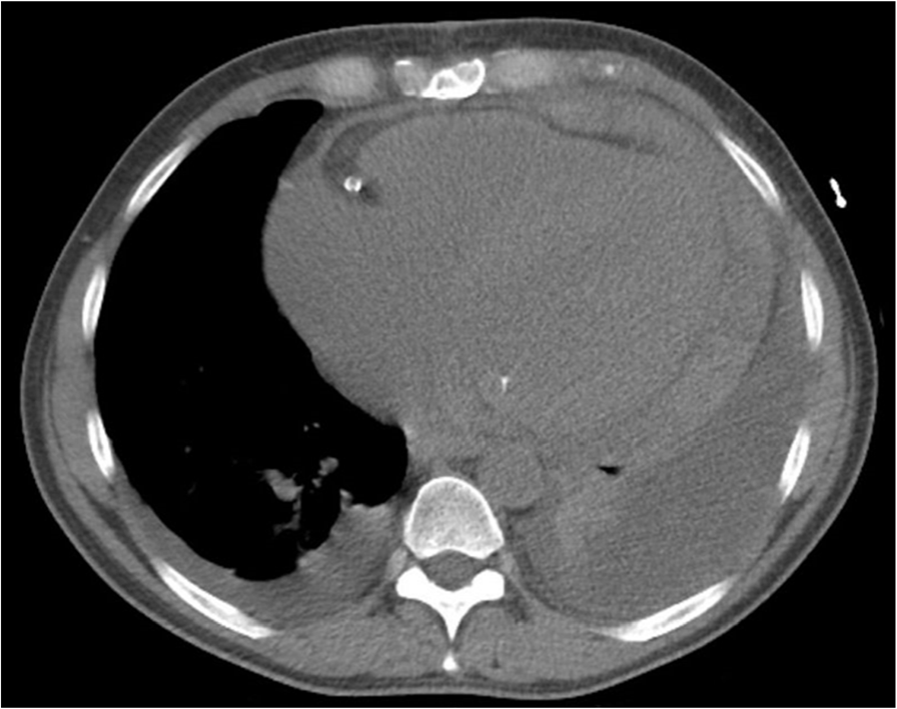
Non-contrast axial image from a dedicated aortic CT through the lower chest, showing hemopericardium in a 43-year-old male who being evaluated for congestive heart failure and low ejection fraction, following aortic valve surgery

Pericardial effusion results when more than 60 mL of fluid accumulates in the pericardial sac. Hemorrhagic pericardial effusion is better characterized on unenhanced CT and measures between 40 and 60 HU (Hounsfield units), compared to less than 10 HU for simple fluid/transudates and between 10 to 20 HU for exudates (Fig. 16). Hemopericardium can occur following left ventricular free wall rupture in myocardial infarction, with ventricular or coronary aneurysmal rupture, in postsurgical patients, in the setting of trauma, and with malignancy [83].

#### Pericardial calcification

**Fig. 17:**
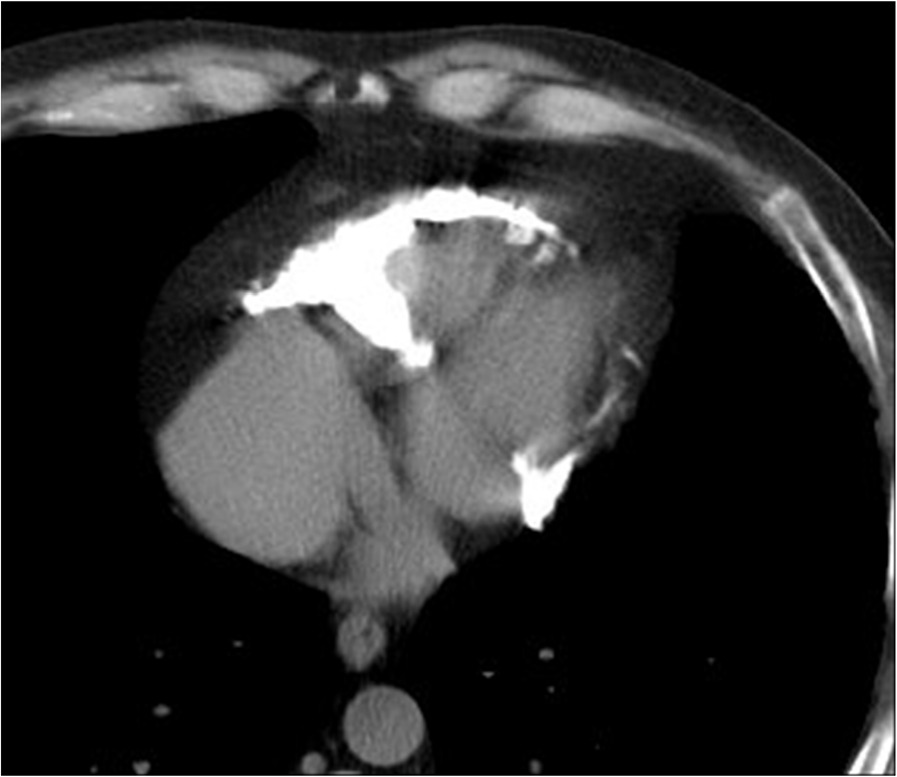
Axial CT image demonstrates chunky pericardial calcification on a non-contrast image, in the AV grooves and bilaterally in a 36-year-old male being investigated for chronic pain

Pericardial calcification commonly results from trauma, infection, hemorrhage, or therapeutic radiation [67]. Traumatic etiologies include surgical exploration during pericardiotomy for cardiac shunt surgery. Tuberculosis and histoplasmosis as infectious etiologies are now less commonly seen in the developed world. Calcification resulting from radiation will confirm to the radiation port used. The calcification may be a focal plaque or a thin curvilinear focus following the cardiac contour. Lesions are usually 1 to 2 mm thick but can increase to 1 to 2 cm in thickness in chronic cases. Pericardial calcification is most commonly found in the atrioventricular (AV) groove and in the lower and diaphragmatic portions of the pericardium (Fig. 17). It is most commonly a global process, involving both sides of the heart. Myocardial calcification tends to localize to the left heart (where the myocardium is thickest) and is thinner than pericardial calcification [67]. Up to 28% of patients with constrictive pericarditis have pericardial calcification, but the converse is not true [64]. Pericardial calcifications may be present without any physiological compromise to the heart. If pericardial calcification is seen on a chest CT, evaluation of the images for signs of constriction is recommended, with consideration for ECHO and or cardiac MRI to further evaluate [84, 85].

### Filling defects in the cardiac chambers

#### Thrombi

**Fig. 18:**
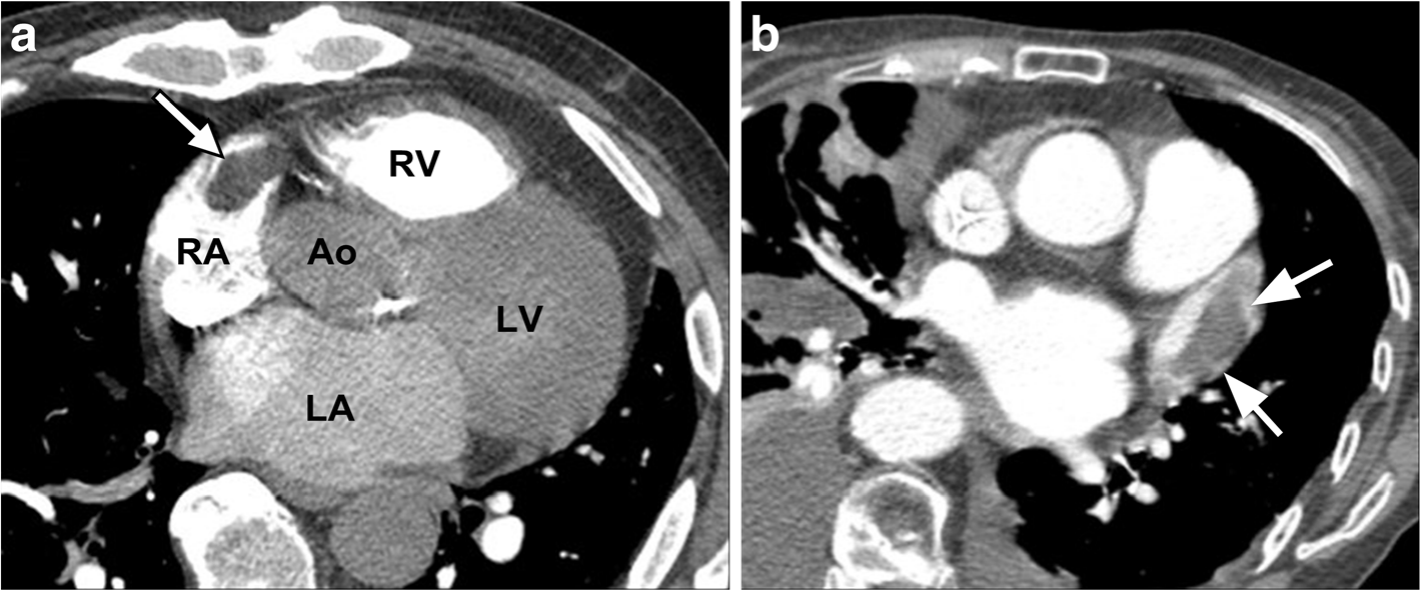
**a** Axial CT image demonstrating thrombus (black arrow) in the right atrium in a 73-year-old patient who had CT to evaluate for pulmonary embolus. **b** Axial CT image demonstrates thrombus within the appendage of the left atrium (white arrows) of another patient who had atrial fibrillation who was being evaluated for pleural effusions

Thrombus is the most common filling defect found in cardiac chambers with a predilection for the left atrial appendage and left ventricular apex (Fig. 18) [85]. In sinus rhythm, left atrial appendage thrombi are usually associated with mitral valve disease or left atrial chamber and appendage dysfunction. The incidence of left atrial thrombi is significantly higher in patients with atrial fibrillation and mitral valve disease [86]. Right atrial thrombus is more likely to occur in the presence of central venous catheters. Patients with wall motion abnormalities, such as left ventricular aneurysm, are at increased risk for left ventricular thrombi. Patients with prosthetic cardiac valves and pacemakers are also at increased risk. Intracardiac thrombi are complicated by arterial embolism in up to 20% of cases, and therefore early identification with subsequent therapy is important [86]. On CT imaging, thrombi typically do not enhance but chronic thrombi can appear heterogeneous with peripheral fibrous capsule formation or they may calcify [85]. Cardiac MRI can help distinguish thrombi from masses, such as tumor thrombus or myxomas.

#### Myxomas

**Fig. 19:**
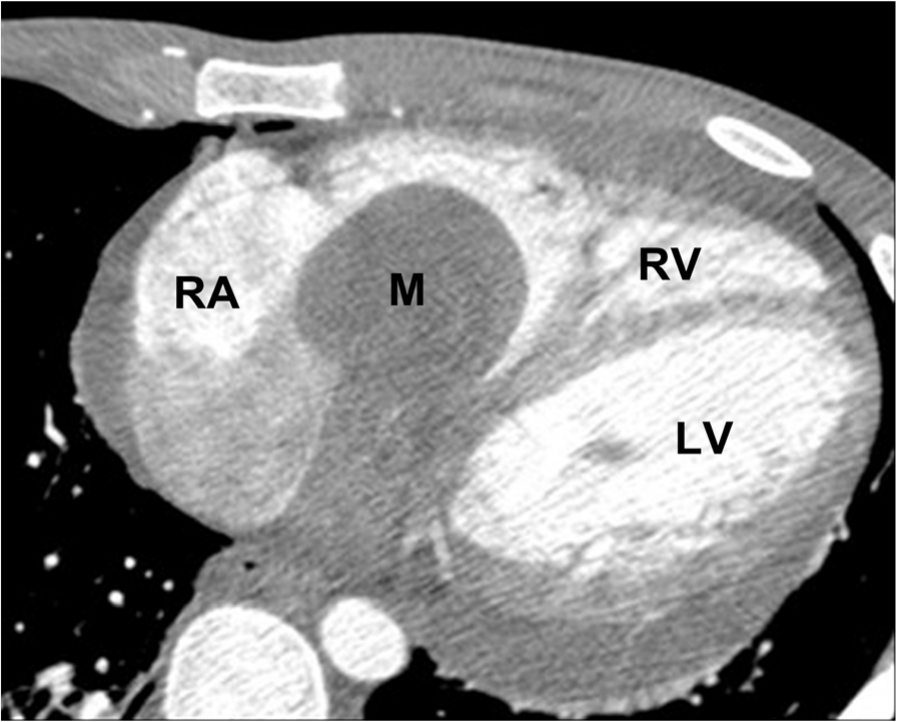
Axial contrast-enhanced CT shows a pedunculated myxoma in the right atrium, which extends through the tricuspid valve into the right ventricle. *RA* right atrium, *M* myxoma, *RV* right ventricle, *LV* left ventricle

Cardiac myxomas are the most common primary neoplasm and account for about half of all primary benign cardiac tumors [87, 88]. The majority (75%) arise within the left atrium, typically at the interatrial septum near the limbus of the fossa ovalis. Most are sporadic, but occasionally there is a familial predisposition or an associated clinical complex (e.g., Carney complex). Myxomas may be asymptomatic or present with cardiac obstructive symptoms, embolic phenomena, and constitutional symptoms. On contrast-enhanced CT imaging, myxomas are usually lower in attenuation (occasionally the same attenuation as myocardium) within the contrast filled cardiac chamber (Fig. 19). They can arise from any endocardial surface and their attachment can be difficult to determine by CT. Myxomas may be heterogenous in attenuation due to calcification (particularly in the right heart), areas of hemorrhage, thrombus, or hemosiderin deposition. Origin and size can sometimes be used to help differentiate myxomas from thrombus with myxomas being larger, and often arising from the fossa ovalis as opposed to the left atrial appendage [89].

### Malignant cardiac masses

#### Metastases

**Fig. 20:**
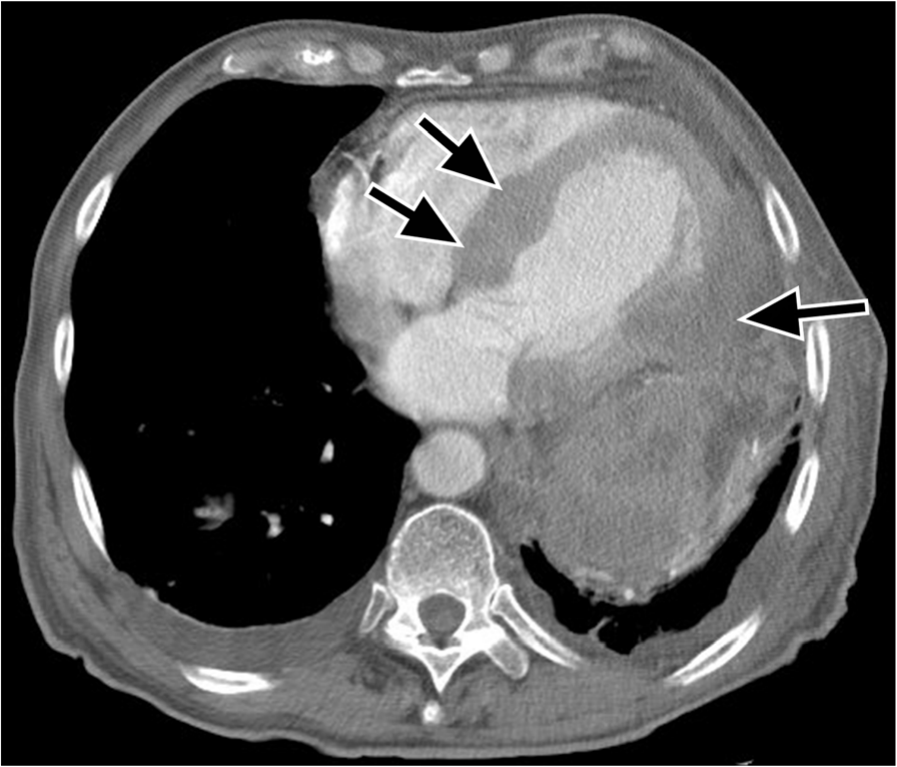
Axial CT with contrast demonstrates nodules and masses (black arrows) in the interventricular septum and the left ventricular free myocardial wall, in a 78-year-old male patient with metastatic malignant melanoma. There is also a large necrotic lymph node in the left mediastinum adjacent to the cardiac implants

Metastases to the heart and pericardium are 20 to 40 times more common than primary cardiac malignancies and are generally associated with a poor prognosis [90]. Cardiac metastases are more frequently carcinomas than sarcomas, with prevalences of between 1.5 and 25% reported for patients with malignant tumors [91]. Due to their proximity to the heart, many metastases originate in the lung or breast, but all types of malignant disease can involve the heart with an increased propensity reported for melanoma [91]. Tumors involve the pericardium and heart by one of four pathways (direct contiguous spread, hematogenous dissemination, transvenous extension, or retrograde lymphatic invasion). Metastases to the heart and pericardium can manifest as a pulmonary or mediastinal mass with direct invasion, as myocardial masses (due to hematogenous spread) as a central mass extending into the left atrium with venous extension), or as a pericardial effusion or nodularity (due to lymphatic extension) [92]. Direct extension can occur with bronchial, breast, esophageal, and thymic neoplasms due to proximity of these tumors. With hematogenous metastases in the heart and pericardium, spread can occur via the coronary arteries, and there is usually evidence of hematogenous metastases in other organs (Fig. 20). Transvenous extension of tumor thrombus can occur in the right atrium through the superior and inferior vena cava, or into the left atrium via the pulmonary veins.

#### Primary cardiac tumors

**Fig. 21:**
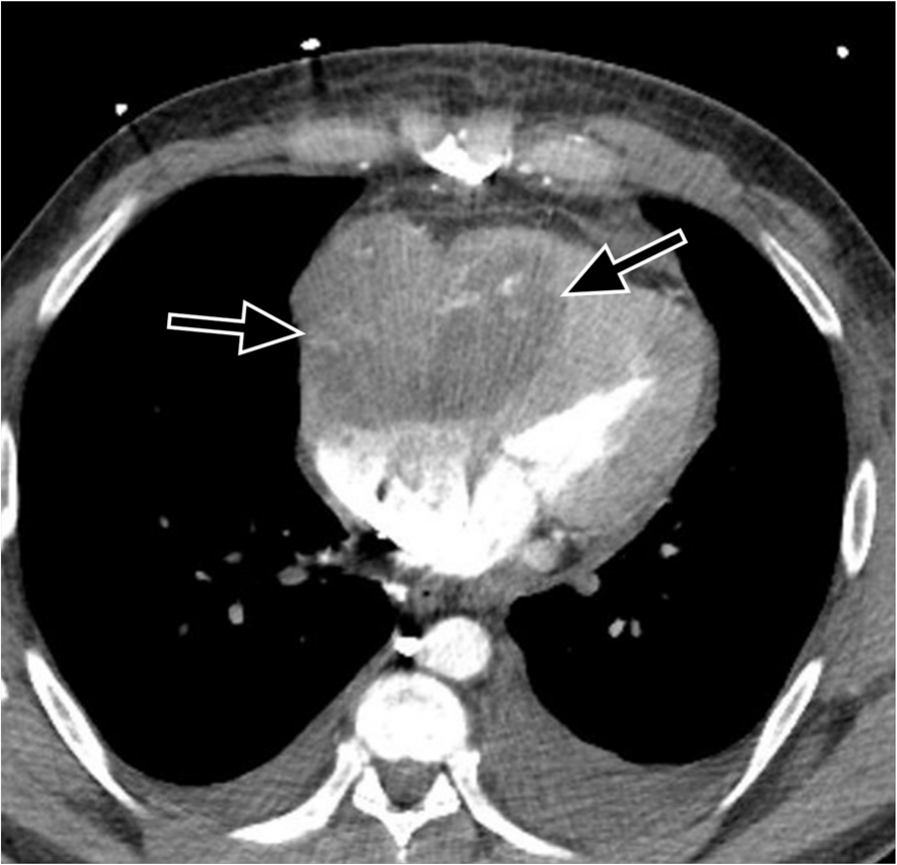
Axial contrast-enhanced CT demonstrates a mass (black arrows) involving the right atrium and ventricle, (later confirmed to be angiosarcoma by histology) on axial contrast enhanced CT and reformatted images in a 27-year-old female being investigated for possible pulmonary embolism (shortness of breath and dizziness)

Primary cardiac tumors are rare with a reported incidence of less than 0.02 to 0.056% with pericardial tumors being even rarer [93]. Primary malignant cardiac tumors account for 25% of primary cardiac tumors [91]. Primary cardiac neoplasms are frequently asymptomatic until they are large and produce non-specific symptoms. Before the advent of cross-sectional imaging, primary cardiac tumors were rarely diagnosed before death. Now, they are found in the living and characterization has become important in directing their management. The most common primary tumor in adults is angiosarcoma [94]. Angiosarcoma predominantly presents in middle-aged male patients, with a slight preponderance reported in males. Angiosarcomas usually involve the right atrium with right heart failure. On CT, these tumors manifest as a discrete mass protruding into a cardiac chamber or as a diffuse infiltrating mass with areas of necrosis (Fig. 21) [91]. These highly vascular tumors exhibit areas of necrosis and pericardial invasion leads to hemorrhagic pericardial effusion and or cardiac tamponade. The prevalence of metastatic disease is reported at 66 to 89% and the prognosis is poor.

Rhabdomyosarcoma is the most common primary cardiac tumor in infants and children, with a slight male predominance. Rhabdomyosarcomas can be multiple, are more likely to occur on the valves, and can occur in all chambers, with variable presentation. They tend to involve the myocardium and can invade the pericardium with nodular masses, rather than diffuse sheet-like thickening. The other sarcomas (undifferentiated, leiomyosarcoma, fibrosacroma, and osteosarcoma) typically involve the left heart chambers and cause left heart failure [94]. Low attenuation components in a mass arising typically from the posterior wall of the left atrium may be seen with leiomyosarcomas, while chondroid or osteoid elements may be seen with osteosarcomas. Unfortunately, delayed diagnosis leads to a poor prognosis with an average survival of 1 year. Cardiac lymphoma is defined as one that is mostly confined to the heart or pericardium (to distinguish it from invasive non-Hodgkin’s lymphoma) and almost all primary cardiac lymphomas are aggressive B cell lymphomas. Unlike other primary cardiac malignancies, primary cardiac lymphoma has a favorable response to chemotherapy. They are more frequent in the right atrium and pericardial effusion is a common feature, and sometimes the only finding visible on imaging. Findings of primary cardiac lymphoma on CT are non-specific.

CT provides good soft tissue detail and can depict calcification, ossification or fat, or make a tissue diagnosis in the case of lipomas. However, cardiac MR imaging has the added advantage of providing functional information and utilizes multiple planes which can help pre-therapy planning [95, 96].

## Conclusion

Cardiac disease and pathology are commonly seen on thoracic CT performed for other indications. Relatively common and significant entities include filling defects due to thrombus or myxoma. Other not uncommonly encountered entities include small left to right shunts, the sequelae of prior MI (such as ventricular fat or calcium deposition), or pericardial constriction. Rarer but significant entities described include coronary artery fistula or aneurysm, anomalous coronary artery course, left ventricular aneurysm, or pseudoaneurysm and malignant cardiac tumors (metastases). Radiologists should be aware of the imaging features of these cardiac diseases encountered on thoracic CT, as some will require specific further referral or diagnostic testing. After completing this article, the reader should be familiar with clinically significant cardiac findings seen on chest CT examinations and be confident to direct the next management steps.

## Abbreviations

ARVC/D: Arrhythmogenic right ventricular cardiomyopathy/dysplasia
ASD: Atrial septal defects
AV: Atrioventricular
BAV: Bicuspid aortic valve
CAF: Coronary artery fistula
CHD: Congenital heart disease
COA: Aortic coarctation
CT: Computed tomography
DCM: Dilated cardiomyopathy
ECG gated: Electrocardiographically gated
ECHO: Echocardiography
HCM: Hypertrophic cardiomyopathy
LA: Left atrium
LAD: Left anterior descending
LCX: Circumflex
LV: Left ventricle
LVE: Left ventricular enlargement
LVH: Left ventricular hypertrophy
LVOT: Left ventricular outflow tract
MDCT: Multi-detector row CT
MRI: Magnetic resonance imaging
PDA: Patent ductus arteriosus
RV: Right ventricle
RVOT: Right ventricular outflow tract
SAM: Systolic anterior wall motion
SOVA: Sinus of Valsalva aneurysm
SVC: Superior vena cava
VSD: Ventricular septal defects
